# Relative performance of hybrid nestlings in *Ficedula* flycatchers: a translocation experiment

**DOI:** 10.1002/ece3.472

**Published:** 2013-01-09

**Authors:** Niclas Vallin, Yuki Nonaka, Jue Feng, Anna Qvarnström

**Affiliations:** Animal Ecology/Department of Ecology and Genetics, Evolutionary Biology Centre, Uppsala UniversityNorbyvägen 18D, SE-752 36, Uppsala, Sweden

**Keywords:** Environmentally dependent hybrid fitness, genetic incompatibility, hybridization, life history, post-zygotic isolation, speciation

## Abstract

Ecological speciation predicts that hybrids should experience relatively low fitness in the local environments of their parental species. In this study, we performed a translocation experiment of nestling hybrids between collared and pied flycatchers into the nests of conspecific pairs of their parental species. Our aim was to compare the performance of hybrids with purebred nestlings. Nestling collared flycatchers are known to beg and grow faster than nestling pied flycatchers under favorable conditions, but to experience higher mortality than nestling pied flycatchers under food limitation. The experiment was performed relatively late in the breeding season when food is limited. If hybrid nestlings have an intermediate growth potential and begging intensity, we expected them to beg and grow faster, but also to experience lower survival than pied flycatchers. In comparison with nestling collared flycatchers, we expected them to beg and grow slower, but to survive better. We found that nestling collared flycatchers indeed begged significantly faster and experienced higher mortality than nestling hybrids. Moreover, nestling hybrids had higher weight and tended to beg faster than nestling pied flycatchers, but we did not detect a difference in survival between the latter two groups of nestlings. We conclude that hybrid *Ficedula* nestlings appear to have a better intrinsic adaptation to food limitation late in the breeding season compared with nestling collared flycatchers. We discuss possible implications for gene flow between the two species.

## Introduction

A major general goal in speciation research is to investigate the mechanisms leading to population divergence and reproductive isolation (Coyne and Orr 2004; Dieckmann et al. 2004; Price 2007). The development of complete reproductive isolation is in most cases a slow process, which follows a temporal pattern starting with ecological divergence and/or the evolution of traits causing sexual isolation, followed by the buildup of genetic incompatibilities (Coyne and Orr 2004; Dieckmann et al. 2004; Price 2007). However, it is also possible that the evolution of sexual isolation and the buildup of genetic incompatibilities precedes major ecological differentiation and that young species go through niche differentiation when they come into periods of secondary contact (Rundell and Price 2009). To what extent ecological differentiation evolves before or after other sources of reproductive isolation hence remains an open question.

Ecological speciation is defined as the evolution of reproductive isolation as a result of divergent selection between populations exploiting different resources or environments (Schluter 2001; Rundle and Nosil 2005). Much research on ecological speciation therefore aims at estimating the relative performance of alternative phenotypes in their local environments using translocation experiments (Rieseberg and Carney 1998; Schluter 2000; Campbell and Waser 2001; Rundle and Nosil 2005). Life-history traits have been shown to play an important role when populations adapt to local conditions, for example to varying temperature regimes (Samietz et al. 2005; Jensen et al. 2008). Divergence in life-history traits could be of particular importance to local adaptation in phases where individuals are unable to escape suboptimal conditions, for example, during the juvenile phase (Jensen et al. 2008).

In this study, we investigate whether a life-history divergence between two closely related species of birds, collared (*Ficedula albicollis*) and pied flycatchers (*F. hypoleuca*), causes environmentally dependent selection on nestling hybrids. Collared and pied flycatchers are genetically separated and a recent study reveals genetic differentiation indicative of periods of allopatric divergence alternated with periods of secondary contact (Ellegren et al. 2012). The two species still partly overlap in plumage characters (Wiley et al. 2005) and in song (Haavie et al. 2004; Qvarnström et al. 2006) and they hybridize at secondary contact (Qvarnström et al. 2010; Sætre and Sæther 2010). There is genetic incompatibility in accordance with Haldane's rule; female hybrids are sterile, while male hybrids mainly experience a disadvantage in competition over mates (Svedin et al. 2008). There is apparently no ecological isolation caused by feeding habits (Wiley et al. 2007) or migration patterns (Veen et al. 2007), but competition over suitable nest sites causes habitat segregation (Vallin et al. 2012) and thereby reproductive isolation (Vallin and Qvarnström 2011). Whereas collared flycatchers mainly occupy food-rich deciduous forests, pied flycatchers have experienced a shift toward poorer quality coniferous forests (Vallin et al. 2012). Moreover, the two species differ in several life-history traits and in their response to the seasonal change in breeding conditions. Collared flycatchers beg relatively more intensively for food (Qvarnström et al. 2007), and have a higher mass gain under favorable conditions early in the season, but are less robust to harsh conditions late in the season (Qvarnström et al. 2005, 2009).

**Figure 1:**
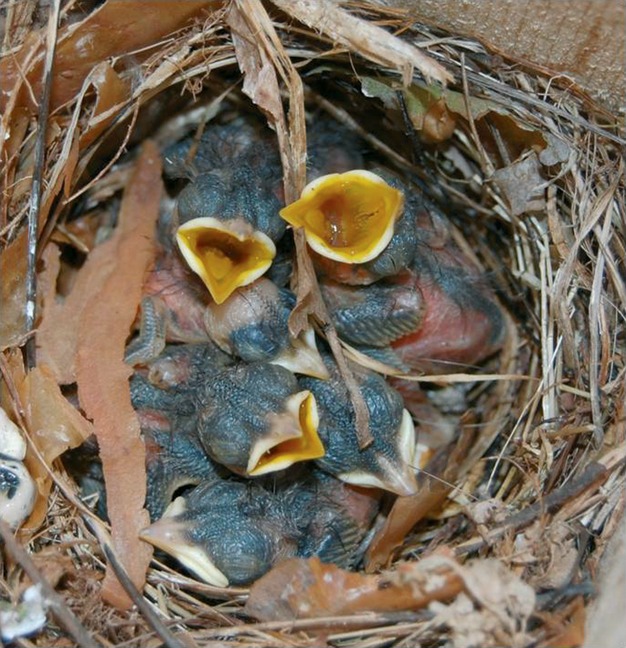
We translocated aviary-born nestling hybrids from crosses between pied and collared flycatchers into the natural nests of the parental species (pied flycatcher nest in the picture) to compare begging intensity, survival, and growth. The experiment was performed on the Swedish island of Öland. (Photo credit: Hanna Arntsen).

In this study, we have three main goals. First, as collared flycatchers are known to breed slightly earlier that pied flycatchers (Vallin et al. 2012) and to inhabit territories containing a high proportion of deciduous trees, whereas pied flycatchers inhabit territories containing a high proportion of coniferous trees (Veen et al. 2010; Vallin et al. 2012), we wanted to test whether hybrid nestlings are reared under intermediate environmental conditions in terms of breeding time and breeding habitat composition. Second, we will investigate whether the relative performance of nestlings produced by heterospecific pairs of *Ficedula* flycatchers is different depending on an environmental factor known to influence the relative performance of nestling pied and collared flycatchers, that is, their parents' timing of breeding. Timing the date of egg laying so that the nestlings are reared under optimal feeding conditions is crucial for maximizing reproductive success in birds (Perrins 1970; Lundberg and Alatalo 1992) and food availability generally declines across the breeding season in the habitats inhabited by the flycatchers (Veen et al. 2010). The above questions were addressed using long-term breeding data from a natural hybrid zone between pied and collared flycatchers. Third, we performed a translocation experiment to compare the relative fitness of purebred and hybrid nestlings under identical environmental conditions. On the basis of the results from previous cross-fostering experiments (Qvarnström et al. 2005, 2007, 2009), we expected hybrid offspring to display intermediate life-history traits in terms of begging behavior and growth rate compared to the two parental species. As mentioned above, much research on ecological speciation aims at estimating the relative performance of alternative phenotypes in their local environments, but this type of experiments are generally not performed with birds. This is because birds have a high ability to move and are difficult to keep in semi-natural enclosures. In this study, we circumvent this problem by the translocation of hybrid nestlings into nests belonging to the two parental species (Fig. 1), thereby facilitating a direct comparison of their performance in relation to purebred nestlings.

## Materials and Methods

### Collection of long-term data

Data on life-history traits were collected from 2002 to 2010 from mixed populations of collared and pied flycatchers breeding on the Baltic island of Öland (57°10′N, 16°58′E). Each year, records are kept of laying date, hatching date, clutch size, and fledging success of breeding birds. All breeding individuals and their nestlings are caught yearly whereupon we ring them, collect blood samples, and take morphological measurements. We estimated the breeding habitat composition for a subset of breeding records where both parents had been identified as either pied or collared flycatchers. The relative abundance of different tree species around the nest-boxes was estimated using a ‘relascope’, assigning individual trees into three categories based on trunk size and distance from the nest-box (see Veen et al. 2010). We thereafter compared the general habitat composition (proportion of deciduous trees) among the territories occupied by the different pairs using a generalized linear model using binomial errors and a logit link. The models were overdispersed (overdispersion ranging from 11.7 to 16.2) and were corrected by the inclusion of an overdispersion parameter. Reproductive success in terms of the number of fledged offspring in relation to breeding time was analyzed using a generalized linear model with Poisson distribution and log link. Because the mean laying date differs between years, we used a standardized timing of breeding in our analyses, which are the residuals from an ANOVA with year as a factor and breeding date as the response variable. We used Wilcoxon signed-rank tests to compare potential differences in breeding time (i.e., laying date of first egg) between con- and heterospecific pairs.

### Cross-fostering experiment

**Figure 2:**
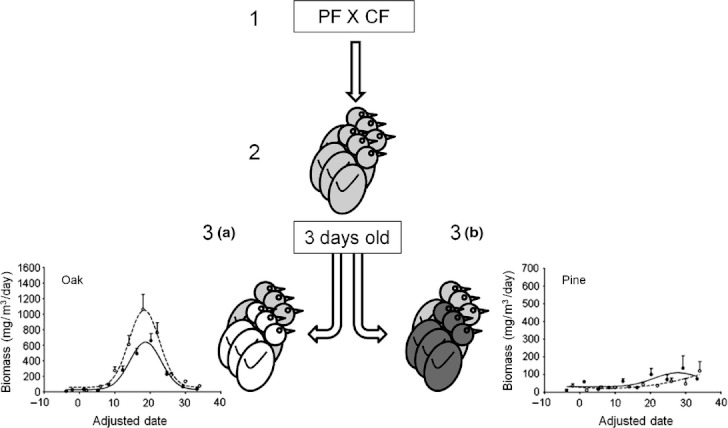
Setup of a *Ficedula* flycatcher cross-fostering experiment performed on the Swedish island of Öland. PF = pied flycatcher, CF = collared flycatcher. 1) Male pied flycatchers and female collared flycatchers were randomly allocated into heterospecific pairs to breed in aviaries. 2) The hybrid offspring were hatched and fed in aviaries until 3) they were 3 days old and translocated to share nests with a) collared flycatcher nestlings, attended by collared flycatcher parents breeding in deciduous forest with a high food peak early in the season (here exemplified by an estimation of caterpillar abundance in oak trees; from Veen et al. 2010), or b) pied flycatcher nestlings, attended by pied flycatcher parents breeding in mixed or coniferous forest with a low food peak late in the season (here, exemplified by an estimation of caterpillar abundance in pine trees; from Veen et al. 2010). The cross-fostering experiments were performed relatively late in the season (see materials and methods).

In 2007 and 2010, we performed artificial breeding experiments in aviaries to obtain F1 hybrid offspring for this study (Fig. 2). Male pied flycatchers and female collared flycatchers were randomly allocated into eight heterospecific pairs in 2007 and additionally 10 in 2010, which bred in separated aviaries (3 × 3 × 2 m). When the hybrid nestlings were 3 days old, we marked them individually by clipping their toenails and placed them into nests of pied and collared flycatchers with coinciding hatching dates. We strived for similar proportions of hybrid versus purebred offspring (mean nr of hybrids and nestling collared flycatchers: 1.8 ± 0.4 SE and 2.1 ± 0.4 SE, respectively; mean nr of hybrids and pied flycatchers: 2.0 ± 0.4 SE and 3.3 ± 0.4 SE, respectively). To avoid increased parental workload by the addition of hybrid offspring, we transferred replaced purebred offspring into other nests. We recorded begging behavior when nestlings were 8 and 9 days old with IR-light cameras (YOKO model YK-3045B, *f* = 3.6-mm broad lens) connected to Digital video cameras (JVC GR-D30). Recordings were made for two 1-h periods on two different mornings. Nestlings were marked individually with water-soluble white out just before recording. A digital videocassette recorder (Panasonic, DVCPRO model AJ-D230) was used to analyze the videotapes. In total, 6468 begging events and 1148 feedings were recorded in for 107 offspring in 20 different nests: 10 attended by collared flycatcher parents and 10 attended by pied flycatcher parents (predated nests, or nests where all nestlings died were excluded). Wilcoxon signed-rank tests were used to compare the mean hatching dates of the experimental nests versus the natural nests, and to compare brood sizes between the experimental nests attended by the two parental species. At each feeding event, nestlings were ranked by the order of when they started to beg, so that the first nestling to beg was ranked nr 1 and so forth. Nestlings begging at the same time got the same ranking, and nestlings that did not beg at all got the last rank. To test whether begging rank influenced the chance of being fed, we used a generalized mixed model (function lmer, package lme4 in R 2.10.1) with being fed (1 or 0) as the response variable and begging rank as explanatory variable. Nestling identity (ring number), rearing nest identity, and year were added as random factors to control for repeated measures on the same individual and variation between nests and years. Logistic regression was applied to compare survival of the nestlings with survival (1 or 0) as the response variable and year as the explanatory variable. To compare the mean begging ranks and weights of hybrids when sharing nests with pied and collared flycatchers, respectively, we used a mixed effects linear model with species identity (hybrid or purebred) and hatching date as fixed effects, and year and rearing nest as random factors to account for variation between years and the non-independence of nestlings within the same nest. Finally, we used the same model to compare weights and growth rates between hybrids reared in the two types of environments. We standardized the hatching date using the residuals from an ANOVA with year as a factor and hatching date as the response variable from all breeding pairs. JMP 9 (SAS Institute, Cary, North Carolina, USA) was used for analyzing the data except where otherwise noted.

## Results

### Comparison of natural rearing environments for hybrids and purebred nestlings

Pied flycatcher breeding territories had a significantly lower proportion of deciduous trees as compared with the territories of heterospecific pairs (*N* = 144, df = 1, χ^2^ = 14.55, *P* < 0.001) and collared flycatcher pairs (*N* = 295, df = 1, χ^2^ = 23.97, *P* < 0.0001). There were no significant differences between heterospecific pairs and collared flycatchers (*N* = 255, df = 1, χ^2^ = 0.70, *P* = 0.40), or between the two types of heterospecific pairs, that is, male collared flycatchers paired to female pied flycatchers and *vice versa* (*N* = 52, df = 1, χ^2^ = 1.35, *P* = 0.24).

Heterospecific pairs showed an intermediate standardized timing of breeding as compared to conspecific pairs: significantly later than collared flycatchers (*N* = 1314, *Z* = 2.874, *P* = 0.004, mean date of egg laying in May = 21.4 ± 0.65 SE and 19.6 ± 0.17 SE for heterospecific and collared flycatcher pairs, respectively) and significantly earlier than pied flycatchers (*N* = 268, *Z* = 2.761, *P* = 0.006, mean date of egg laying in May = 23.3 ± 0.42 SE for pied flycatcher pairs). There were no significant differences in the standardized timing of breeding between the two types of heterospecific pairs (*N* = 95, *Z* = −1.056, *P* = 0.29, mean date of egg laying in May = 21.9 ± 0.82 SE for collared male/pied female, and 20.3 ± 0.96 SE for pied male/collared female).

### Comparison of natural performance of nestlings

**Figure 3:**
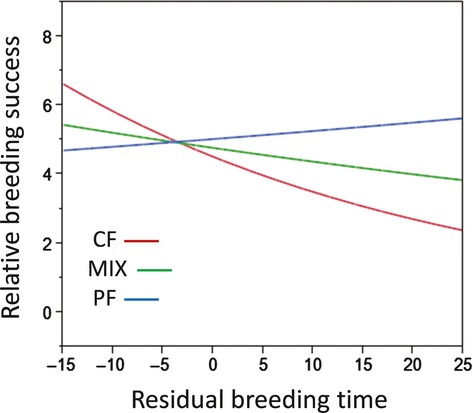
Relative fledging success (trendlines from a generalized linear model with Poisson distribution and log link) of nestlings reared by different types of *Ficedula* flycatcher pairs in relation to their parents' timing of breeding (laying date corrected for variation among years). Nestlings reared by heterospecific pairs (MIX) show an intermediate relative performance across the seasonal compared to pied (PF) and collared (CF) flycatchers. Data collected from nest-box breeding population on the Swedish island of Öland 2002–2010.

We investigated how the seasonal decline in food availability influenced the reproductive success of collared flycatchers, pied flycatchers, and heterospecific pairs. Analyzes of long-term breeding data revealed a significant interaction between pairing type and timing of breeding on the reproductive success of these naturally breeding pairs (*N* = 1305, χ^2^ = 19.56, *P* < 0.0001). As shown before, the reproductive success of collared flycatchers dropped steadily across the breeding season, while the reproductive success of pied flycatchers showed no such trends, rather the opposite (see also Qvarnström et al. 2005). The reproductive success of hetersospecific pairs remained intermediate across the breeding season compared to pure pied or collared flycatcher pairs (Fig. 3). We found no significant difference in fledging success between nestlings raised by a collared male/pied female pair as compared with nestlings raised by a pied male/collared female pair (*N* = 75 χ^2^ = 0.37, *P* = 0.54), after removing the non-significant interaction term between breeding time and pairing type.

### Cross-fostering experiment

**Figure 4:**
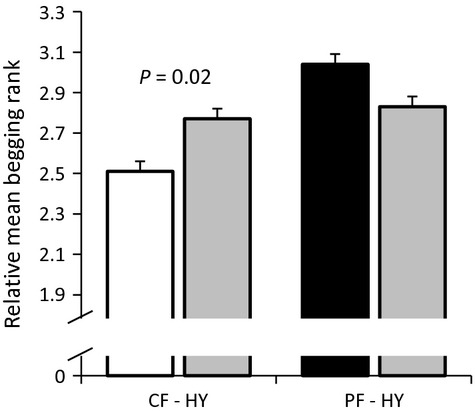
Comparison of relative begging intensity of nestling collared (CF), hybrid (HY), and pied (PF) flycatchers from a cross-fostering experiment performed on the Swedish island of Öland in 2007 and 2010. Hybrid nestlings were matched against purebred nestlings of the two species in artificially created mixed broods and we, here, show the predicted values and S.E. from a mixed effects model (lowest score begs the most). Comparisons were only made between hybrids and pied flycatchers or hybrids and collared flycatchers sharing nests. Significant differences between the nestlings are indicated by the *P*-value given above respective bars.

In order to investigate whether intrinsic differences between hybrid and purebred nestlings influenced their relative fitness during the nestling stage, we artificially created broods containing two types of nestlings. The experiments were carried out relatively late in the breeding season, as revealed by a comparison of the standardized hatching dates of experimental and natural nests (*N* = 1523, *Z* = 2.68, *P* = 0.01, mean standardized hatching date = 3.3 ± 1.1 SE and 0.0 ± 0.13 SE for experimental and natural nests, respectively). We compared the begging behavior, growth patterns, and survival of nestling hybrids sharing nest with purebred nestlings. There were no significant differences in brood size between experimental nests attended by collared or pied flycatcher parents (*N* = 20, df = 1, *Z* = 1.51, *P* = 0.13, mean brood size = 4.9 ± 0.4 SE, and 5.8 ± 0.4 SE for nests attended by collared and pied flycatcher parents, respectively), that is, the begging ranks were on a comparable scale. Begging rank significantly influenced the chance of being fed as revealed by a generalized mixed model (*N* = 6468, df = 1, *Z* = −20.55, *P* < 0.0001, slope = −0.535 ± 0.03 SE), that is, nestlings with a lower begging score were more likely to be fed first. Nestling collared flycatchers begged significantly faster than hybrid nestlings when sharing nests attended by collared flycatcher parents (*N* = 49, df = 1, *F*_1,41.73_ = 5.70, *P* = 0.02, least square means = 2.51 ± 0.61 SE and 2.79 ± 0.61 SE for collared flycatchers and hybrids, respectively, fig. 4). There was a significant effect of breeding time on the mean begging rank in nests shared by hybrid and collared flycatcher nestlings (*N* = 49, *F*_1,7.72_ = 7.17, *P* = 0.03, slope = −0.047 ± 0.02 SE), but no significant interaction between date and type of nestling, that is, both hybrids and collared flycatcher nestlings begged more later in the season. By contrast, there was a non-significant tendency for hybrids to beg faster than nestling pied flycatchers in nests attended by pied flycatcher parents (*N* = 58, *F*_1,47.66_ = 2.90, *P* = 0.10, least square means = 3.07 ± 0.38 SE and 2.79 ± 0.39 SE for pied flycatchers and hybrids, respectively, fig. 4). There was no significant effect of breeding time on the mean begging rank in nests shared by hybrid and pied flycatcher nestlings (*N* = 58, *F*_1, 6.99_ = 2.36, *P* = 0.17).

**Figure 5:**
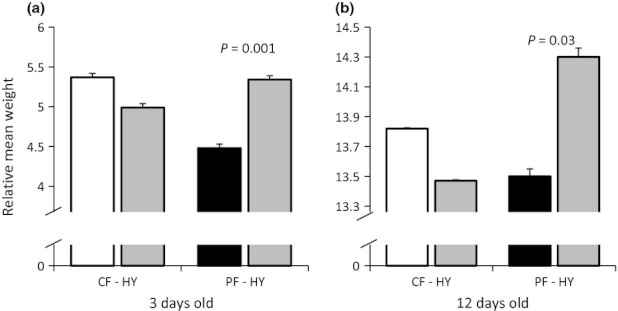
Comparison of relative mean weight in gram of a) 3- and b) 12-day-old nestling collared (CF), hybrid (HY), and pied (PF) flycatchers. Weights were collected from an experiment performed on the Swedish island of Öland in 2007 and 2010 where hybrid nestlings were cross-fostered with either pied or collared flycatchers. Values on y axis are predicted values and S.E. from a mixed effects model, significant differences between the nestlings are indicated by the *P*-value given above respective bars. Comparisons were only made between hybrids and pied flycatchers or hybrids and collared flycatchers sharing nests.

The mass of nestling hybrids did not differ from the mass of nestling collared flycatchers when they were 3 days old (*N* = 44, *F*_1, 35.73_ = 1.27, *P* = 0.26 least squares mean = 5.32 ± 0.35 SE, and 4.97 ± 0.34 SE for collared flycatchers and hybrids, respectively; the variance component for year was negative and dropped from the model) or 12 days old (*N* = 26, *F*_1, 19.08_ = 1.86, *P* = 0.19; least squares mean = 13.83 ± 1.70 SE, and 13.48 ± 1.70 SE for collared flycatchers and hybrids, respectively, fig. 5). However, hybrids had a significantly higher survival rate compared with collared flycatchers (*N* = 49, df = 1, χ^2^ = 3.93, *P* = 0.05), and there was a significant effect of year on survival (*N* = 49, df = 1, χ^2^ = 55.92, *P* < 0.0001). Nestling hybrids raised in pied flycatcher nests were significantly heavier than the nestling pied flycatchers both at the age of 3 days (*N* = 58, *F*_1, 47.95_ = 12.08, *P* = 0.001, least squares mean = 4.47 ± 0.43 SE, and 5.36 ± 0.45 SE for pied flycatchers and hybrids, respectively) and 12 days (*N* = 52, *F*_1, 42.27_ = 4.92, *P* = 0.03, least squares mean = 13.57 ± 1.04 SE, and 14.30 ± 1.05 SE for pied flycatchers and hybrids, respectively, Fig. 5). There was no overall difference in weight gain from day 3 to 12 between nestling pied flycatchers and hybrids (*N* = 52, *F*_1, 41.87_ = 0.11, *P* = 0.74). There was a significant effect of year on survival (*N* = 58, df = 1, χ^2^ = 7.06, *P* = 0.01), but no significant differences in survival between these two types of nestlings (*N* = 58, df = 1, χ^2^ = 0.80, *P* = 0.37). Hybrids reared in the two types of social environments did not differ in weight compared with each other at day 3 (*N* = 44, *F*_1, 14.78_ = 0.52, *P* = 0.48), or day 12 (*N* = 35, *F*_1, 12.23_ = 0.58, *P* = 0.46), and there was no difference in growth rate (*N* = 35, *F*_1, 12_ = 0.00, *P* = 0.99).

## Discussion

In this study, we found that nestlings raised by hybridizing flycatchers experienced intermediate fledging success compared to the two types of purebred nestlings across the breeding season (Fig. 3). Early in the season, nestlings produced by hybridizing flycatchers had a lower fledging success than collared flycatchers, but a higher fledging success than pied flycatchers. Late in the season, nestlings produced by hybridizing flycatchers had a higher fledging success as compared with collared flycatchers, but a lower fledging success than pied flycatchers. In order to disentangle the role of parental effects and intrinsic differences between nestlings, we placed hybrid nestlings in the same nests as collared and pied flycatcher nestlings. This experiment was performed late in the breeding season. We found that nestling F1 hybrids tended to beg faster for food and had a higher weight compared with nestling pied flycatchers, although there was no difference in weight gain in shared nests. By contrast, nestling collared flycatchers started to beg significantly faster and had a lower survival chance compared with nestling hybrids in shared nests. We found no difference in weight between hybrids raised in the two different social environments.

The seasonal changes in relative reproductive success of hybridizing flycatchers in relation to breeding pairs of pied and collared flycatchers may depend on differences in adaptations of both adults and nestlings. We found that the breeding territories of pied flycatcher pairs had a significantly lower proportion of deciduous trees as compared with the territories of heterospecific pairs (see also Vallin and Qvarnström 2011 for a comparison restricted to hybridizing and non-hybridizing male pied flycatchers). The fact that hybridizing flycatchers experienced a lower fledging success than pied flycatchers late in the season may therefore partly be explained by the difference in habitat occupancy because the peak in food abundance is lower, less steep, and occurs later in the season in the mixed forest as compared with deciduous forest (Veen et al. 2010). However, we did not find any indications suggesting that heterospecific pairs breed in different habitats compared with collared flycatchers. Thus, intrinsic differences between nestlings may play an important role in explaining the seasonal changes in relative reproductive success between hybridizing and non-hybridizing collared flycatchers.

Previous cross-fostering experiments imply that genetic differences between nestling pied and collared flycatchers affect their begging behavior and growth (Qvarnström et al. 2005, 2007, 2009). We therefore expected nestling F1 hybrids to have an intermediate begging intensity and growth strategy, which may influence their robustness to harsh conditions. In the experimental nests, we found that nestling collared flycatchers indeed started to beg significantly faster than hybrids in collared flycatcher nests, whereas in pied flycatcher nests, purebred nestlings did not beg faster than hybrids, rather the opposite (Fig. 4). In nests attended by collared flycatchers, both nestling hybrids and nestling collared flycatchers increased their begging frequency as the season was progressing. The begging behavior of nestlings has been shown both theoretically (Godfray 1991) and empirically (e.g., Kilner and Johnstone 1997) to honestly reflect the nestling's need for food. This pattern has also been confirmed in *Ficedula* flycatchers (Rosivall et al. 2005; Qvarnström et al. 2006), where the mean begging rank of an individual nestling influences how many times it is fed by parents and predicts its mass at fledging (Qvarnström et al. 2007). We found that begging rank predicted the likelihood of being fed also in this study and we interpret our results as nestling collared flycatchers having a higher food demand and growth potential, but being more sensitive to harsh conditions compared with hybrid nestlings. This conclusion was further strengthened by the fact that nestling collared flycatchers had a lower survival chance compared with nestling hybrids in shared nests. A relevant question then becomes why we did not find nestling collared flycatchers to experience a relative fitness advantage in the earliest experimental broods. A likely explanation to the lack of this pattern is that all experiments were carried out relatively late in the breeding season when the differences in breeding success are more pronounced between the species (Fig. 3).

The relative performance of nestling F1 hybrids appears to be different in the nests shared with nestling pied flycatchers that were attended by pied flycatcher parents. Nestling F1 hybrids tended to beg faster than nestling pied flycatchers in these nests (Fig. 4), but this difference was not statistically significant, and there was no significant increase in begging intensity as the season proceeded. Nestling hybrids were significantly heavier than nestling pied flycatchers throughout the breeding season, although there were no differences in mass gain between day 3 and day 12 between them (Fig. 5). A potential important factor contributing to these findings might be that the pied flycatchers' territories in coniferous or mixed forests actually provide a more stable environment late in the season compared with deciduous forests (Veen et al. 2010), and/or that the adult pied flycatchers are proficient in locating suitable prey also late in the season. This is also in line with the finding that neither nestling hybrids nor nestling pied flycatchers increased their begging frequency across the season in these nests. In fact, nestling collared flycatchers experience a fitness advantage as compared to nestling pied flycatchers across the whole season in the nests of pied flycatchers (Qvarnström et al. 2005, 2009). However, the fact that we found no significant difference in weight at fledging between hybrids that were fostered in pied flycatcher nests compared with hybrids that had been fostered in collared flycatcher nests implies that the possible effects of differences in habitat choice and parental effort between the two species are small in relation to the effects of the seasonal change in food availability. Thus, intrinsic differences between hybrid and purebred nestlings appear to have a large effect on their relative performance across the breeding season while parental effects appear to be rather minor.

We are not aware of any similar experiments performed in natural bird hybrid zones. The fact that both flycatcher species are generalist insect eaters that mainly differ in life-history traits was an important prerequisite for the possibility to perform this type of experiment. However, it is also important to keep in mind that working within a natural hybrid zone imposes some practical and ethical constraints on our experimental design. We therefore limited the pair-combination in aviaries to male pied flycatcher/female collared flycatcher. On the basis of the long-term analysis of reproductive success in heterospecific pairs, we did not expect the male/female combination to significantly affect the performance of hybrid nestlings. A crucial question then becomes whether being hatched and raised in the aviary until day 3 could have resulted in a survival advantage of hybrid nestlings fostered into natural collared flycatcher nests. However, there was no difference in mean mass between hybrid nestlings and collared flycatcher nestlings when they were placed in the same nest. We also consider it more likely that the aviary conditions if anything would result in a slight disadvantage due to a less varied diet. Due to a large amount of previous cross-fostering studies performed in this study system, we know that cross-fostering *per se* has no significant effect on nestling performance. This is because parents do not discriminate between their own and cross-fostered young when feeding them (see Qvarnström et al. 2005, 2007, 2009 for experiments performed on Öland, and e.g., Cichoń et al. 2006; Pitala et al. 2007; Ruuskanen et al. 2009 for experiments performed on Gotland).

The apparently intermediate adaptations of nestling hybrids mean that they can have both a fitness advantage as well as a fitness disadvantage compared to the two parental species depending on changes in environmental conditions in time and space. Hence, late in the breeding season (or in years with low food availability), we would expect a relatively high production of hybrid nestlings due to a higher tolerance to poor conditions in hybrid nestlings compared with collared flycatcher nestlings. Early in the breeding season (or in years with abundant food availability), we would expect the opposite. In Darwin's finches, selection on medium ground finches (*Geospizia fortis*), cactus finches (*G. scandens*), and their hybrids changes are depending on fluctuations in environmental conditions (e.g., Grant and Grant 2002). As there are no indications of genetic incompatibilities between those finches, species integrity solely depends on ecological conditions and on assortative mating (see Arnold and Martin 2010 for a review on variation in hybrid fitness across environments in other taxa). As compared to Darwin's Finches, *Ficedula* flycatchers have reached a later stage in the speciation process. Does this mean that ecologically based isolation is irrelevant in the flycatcher case? We argue that there are several reasons for assuming that ecologically based reproductive isolation is likely to play a crucial role for the speciation process also in *Ficedula* flycatchers. First, the two species started to diverge approximately 1–2 million years ago, and were probably periodically isolated in separate glacial refuges during the Pleistocene (Sætre et al. 2001; Ellegren et al. 2012). Ecologically based selection on hybrids may therefore have been important in determining the rate of gene flow when the two flycatcher species experienced periods of sympatry in the past. Second, we have recently shown that a feedback loop between ecological and reproductive character displacement is causing fast further ongoing divergence between the two *Ficedula* flycatchers (Vallin et al. 2012) and affects pre-zygotic isolation through habitat segregation (Vallin and Qvarnström 2011). Periods of secondary contact can hence speed up ecological divergence (niche separation) and therefore also strengthen environmentally dependent selection on hybrids. There is an interesting possibility that ecologically driven divergence in life-history traits between the two *Ficedula* species is linked with the evolution of genetic incompatibility; if the most divergent strategies are favored by selection (Vallin et al. 2012), the probability of genetic incompatibilities between the species will also be enhanced as the overall genetic differences are increasing.

In addition to pre-zygotic barriers such as plumage and song (Svedin et al. 2008) and ongoing habitat segregation (Vallin and Qvarnström 2011), there is a high total level of post-zygotic isolation between pied and collared flycatchers due to low fertility of hybrids (Wiley et al. 2009). As female hybrids are totally sterile (Svedin et al. 2008), only back crossing with partially fertile male hybrids can lead to gene flow and there is a low production of hybrid males to start with. Female collared flycatchers, for example, rarely pair with male pied flycatchers and when they do so, they often breed relatively late and show a high proportion of purebred offspring in their nests resulting from extra pair copulations (Veen et al. 2001). However, our results implying that hybrid nestlings experience a survival advantage compared to collared flycatchers late in the season provide at least a partial explanation to why there still appears to be ongoing gene flow between the two species (Ellegren et al. 2012). We would like to stress that the strongly reduced fertility of hybrid males (Ålund et al., submitted manuscript) means that hybridization is never an adaptive option to mate with a conspecific male for female collared or pied flycatchers.

In summary, in this study, we have shown that a life-history divergence between two closely related species can induce environmentally dependent relative fitness of hybrid offspring. Thus, the impact of the environment on the direction and level of gene flow between parental species need not be limited to effects of pre-zygotic isolation once genetic incompatibilities are present.
